# An optimized and validated protocol for the purification of PDGFRα+ oligodendrocyte precursor cells from mouse brain tissue via immunopanning

**DOI:** 10.1016/j.mex.2023.102051

**Published:** 2023-02-02

**Authors:** Julia Macintosh, Mackenzie A. Michell-Robinson, Xiaoru Chen, Daryan Chitsaz, Timothy E. Kennedy, Geneviève Bernard

**Affiliations:** a Department of Neurology and Neurosurgery, McGill University, Montreal, QC, Canada; b Child Health and Human Development Program, Research Institute of the McGill University Health Center, Montreal, Quebec, Canada; c Neuroimmunology Unit, Montreal Neurological Institute, Montreal, Quebec, Canada; d Department of Pediatrics, McGill University, Montreal, Quebec, Canada; e Department of Human Genetics, McGill University, Montreal, Quebec, Canada; f Department Specialized Medicine, Division of Medical Genetics, McGill University Health Center, Montreal, Quebec, Canada

**Keywords:** Immunopanning, Oligodendrocyte precursor cells, Mouse primary cells, Oligodendrocyte purification, *in vitro*, Immunopanning oligodendrocyte lineage cells from mouse brain

## Abstract

Immunopanning is an efficient and reliable method for isolating primary cells from rodent brain tissue, making it a valuable tool for researchers interested in *in vitro* glial models. Here, we present an immunopanning protocol optimized for the isolation of Platelet-Derived Growth Factor Receptor Alpha positive (PDGFRα+) oligodendrocyte precursor cells (OPCs) from mouse brain tissue that results in a high yield of pure OPCs from minimal quantities of starting tissue.

• The protocol presented here is optimized for a PDGFRα-dependent selection of mouse OPCs using a commercial antibody, accounting for the relatively weaker adhesion of OPCs to the anti-PDGFRα plate as compared to other oligodendrocyte lineage markers (e.g., MOG).
• A modified papain digestion step, with 95% O_2_/5% CO_2_ gas that is humidified prior to perfusion, significantly enhances the yield of dissociated cells and final yield of OPCs.
• Isolating OPCs at the PDGFRα+ stage permits the expansion of cells in culture, facilitating studies using transgenic mice, and enables studies on the development of the oligodendrocyte lineage without the spatial and temporal complexity of *in vivo* studies.

## Graphical abstract

**Figure:**
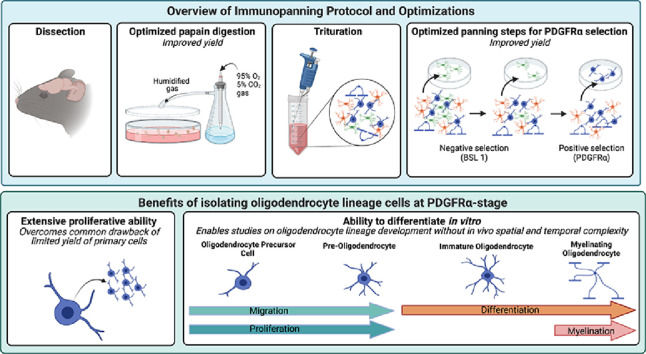

Specifications Table

**Table:** 

Subject area	Neuroscience

More specific subject area:	*In vitro studies of primary murine oligodendroglia cells*
Name of your method:	*Immunopanning oligodendrocyte lineage cells from mouse brain*
Name and reference of original method:	Emery, B., & Dugas, J. C. (2013). Purification of oligodendrocyte lineage cells from mouse cortices by immunopanning. *Cold Spring Harbor protocols*, *2013*(9), 854–868. https://doi.org/10.1101/pdb.prot073973
Resource availability:	N.A.

## Method details

### Introduction

Various techniques have been developed for the isolation of oligodendrocyte precursor cells (OPCs) and oligodendrocytes (OLs) from rodent brain tissue, such as magnetic-activated cell sorting (i.e., MACS), fluorescence-activated cell sorting (i.e., FACS), and physical separation by shear force from mixed glial cultures (i.e., shake-off method) [1], [2], [3], [4], [5]. While many of these techniques have proven successful for the culture of OL lineage cells from rat brains, culturing mouse OPCs remains a challenge. While rat OPCs and astrocytes stratify in mixed glial cultures and can be separated when exposed to shear force, mouse OPCs are difficult to detach and isolate from astrocytes, resulting in low yield or impure cultures [6]. Furthermore, MACS and FACS have traditionally relied on cell-surface antigens that are highly expressed in rat OPCs but show relatively lower or less specific expression in mouse cells, leaving these techniques flawed for OPC isolation from mouse brain tissue [6,7]. Consequently, many *in vitro* studies of the oligodendrocyte lineage have relied on rat cells.

Given the preponderance of transgenic models in mice among rodents and their widespread use in studies of brain development, function, and disease, a reliable method to isolate primary OPCs from the mouse brain is of great significance. Equally important, such a method must yield adequate numbers of healthy cells in order to pursue detailed *in vitro* studies from minimal starting material. Transgenic breeding programs often yield a genotype of interest in ≤25% of pups. It is often the case that a standard litter (∼6 pups) from two heterozygous breeders yields just one pup having a target genotype. Therefore, the challenge of efficiently isolating healthy mouse OPCs in sufficient numbers is of equal or even greater importance than the challenge of isolating the cells themselves.

Immunopanning is a reliable and sensitive method for the isolation of mouse OL lineage cells, whereby a single cell suspension derived from dissociated brain tissue is sequentially added onto cell culture dishes containing absorbed materials (e.g., lectin, antibody). The cell suspension is first added to negative selection plates, depleting unwanted cell types from the mixture, and finally onto a positive selection plate, to retain the desired cell type [8]. The original published protocol, Emery & Dugas (2013), summarizes immunopanning for the isolation of oligodendrocyte lineage cells at various stages of development (e.g., PDGFRα selection for OPCs, O4 selection for immature oligodendrocytes, MOG selection for mature oligodendrocytes) [8]. Here, we present an improved immunopanning protocol for the isolation of PDGFRα+ OPCs from mouse brain, which accounts for both the poor recovery of these cells following tissue dissociation and the relatively weak binding of OPCs to anti-PDGFRα plates during the PDGFRα-dependent selection, resulting in a high-yield and pure cultures.

## Materials and reagents

Dissection and Papain Dissociation Reagents

• Earle's Balanced Salt Solution (EBSS; Wisent, 311–210-CL)
• Papain (Worthington, LS003126)
• Papain buffer
  ○ MgSO_4_
  ○ EGTA (1 M)
  ○ Glucose
  ○ NaHCO_3_ (1 M)
  ○ EBSS (10X) stock
• L-Cysteine (Sigma, C7477)
• 10X papain inhibitor, 6X papain inhibitor
  ○ Bovine Serum Albumin (BSA; Sigma, A3912)
  ○ Ovomucoid (Worthington, LS003086)
  ○ D-PBS with Ca^2+^/Mg^2+^ (ThermoFisher, 14287080)
  ○ NaOH
• DNase I
  ○ DNase I (CedarLane, LS002007)
  ○ EBSS (Wisent, 311210CL)

Immunopanning Reagents

• BSL 1 (BioLynx, VECTL1100)
  ○ HEPES Buffered Saline
    ■ NaCl
    ■ Na_2_HPO_4_
    ■ HEPES
    ■ NaOH
    ■ CaCl_2_
    ■ NaN_3_
• Goat anti-rat IgG (Jackson ImmunoResearch, 115–005–167)
• Tris–HCl (50 mM) stock solution
• Phosphate-buffered saline (PBS; ThermoFisher, 10010023)
• Anti-PDGFRα antibody (BD Pharmigen, 558774)
• Immunopanning buffer
  ○ Bovine Serum Albumin (Sigma, A3912)
  ○ Insulin from bovine pancreas (Sigma, I6634)
  ○ D-PBS with Ca^2+^/Mg^2+^ (ThermoFisher, 14287080)

Trypsinization Reagents

• Trypsin from bovine pancreas (Sigma, T9935)
• EBSS (Wisent, 311210CL)
• EBSS (10X) stock
  ○ NaCl
  ○ KCl
  ○ NaH_2_PO_4_•H_2_O
  ○ Glucose
  ○ Phenol red (0.5%) (Sigma, P0290)
• Fetal Bovine Serum (FBS; Wisent, 098–150)

Cell culture reagents

• Poly-D-Lysine (PDL) hydrobromide (Sigma, P8099)
  ○ Borate buffer
    ■ H_3_BO_3_
    ■ Na_2_B_4_O_7_
• Cell culture media
  ○ Dulbecco's Modified Eagle's Medium (DMEM; Wisent, 319–005-CL)
  ○ SATO supplement
    ■ BSA (Sigma, A3912)
    ■ Transferrin (Sigma, T1147)
    ■ Putrescine (Sigma, P7505)
    ■ Progesterone (Sigma, P8783)
    ■ Sodium selenite (Sigma, S5261)
  ○ Insulin (0.5 mg/mL) stock solution
  ○ Penicillin-Streptomycin (Wisent, 450–201-EL)
  ○ Sodium pyruvate (Gibco, 11360070)
  ○ GlutaMAX (Gibco, 35050061)
  ○ N-Acetyl-L-Cysteine (Sigma, A7250)
  ○ Forskolin (Sigma, F3917)
    ■ Dimethyl sulfoxide (DMSO; Fisher, BP231–100)
  ○ D-Biotin (Sigma, B4639)
  ○ Trace Elements B (Fisher Scientific, MT99175Cl)
  ○ B-27 without vitamin A (ThermoFisher, 12587010)
  ○ Recombinant human PDGF-AA (Preprotech, 100–13A)
  ○ Recombinant human bFGF (Preprotech, 100–18B)
  ○ Triiodothyronine (T3, 4 µg/mL) (Sigma, T6397)
• Trypsin/EDTA (optional, for passaging) (Wisent, 325–042-CL)

Materials and equipment

• Ready Warm Hot plate (LW scientific, FWL-04PL-BTD3)
• 500 mL vacuum flask
• Tubing (∼0.5 cm wide)
• Connectors for tubing
• 500 mL flask stopper with one-hole
• 10 cm petri dishes (Fisher, FB0875713)
• Petri lid
• 95% O_2_/5% CO_2_ gas tank (MEGS Air liquide, A0490943)
• 40 µM cell strainer, DNase/RNase free, non-pyrogenic, sterile (VWR, 76327–098)
• Syringe filter (0.22 µM) (Milipore, SLGV033RS)
• 50 mL Syringe with Luer-Lok tip (BD, 309653)
• 15 mL, 50 mL conical tubes (Sarstedt, 62.554.205 and 62.547.205)
• 2 mL, 5 mL, 10 mL, 25 mL serological pipettes (Sarstedt, 86.1252.001, 86.1253.001,
  86.1254.001 and 86.1685.001)
• 20 µL, 200 µL, 1000 µL pipette tips
• 10 cm cell culture dishes (Sarstedt, 83.3902)
• 6-well (Sarstedt, 83.392O) or 12-well cell culture dishes (Sarstedt, 83.392) (optional, for immunocytochemistry)
• Coverslips (Pre-Treated German Glass Coverslips 15 mm #1; Electron Microscopy Sciences) (optional, for immunocytochemistry)
• Dissection kit: large scissors for decapitation, small scissors, forceps
• Razor Blade (VWR, 55411–055) or scalpel (FisherScientific, 08–927–5F)
• Tabletop centrifuge with tube adaptors for 15 mL and 50 mL tubes
• Biological Safety Cabinet
• Cell culture CO_2_ incubator set to 10% CO_2_
• Water bath, set to 37°C

## Method workflow

A note on planning experiments: The protocol described here is sufficient for processing three P6 mouse brains which yields an average of 6 × 10^6^ PDGFRα+ OPCs in the final step. These are either collected directly or further expanded *in vitro* for downstream testing. To increase throughput, simply coat additional plates and prepare additional reagents.

Preparation the day before immunopanning

1. Coat positive selection plate with secondary antibody.
  a. Dilute 30 µL of anti-rat IgG (1.3 mg/mL) (Jackson ImmunoResearch) in 10 mL of Tris–HCl (50 mM, pH 9.5).
  b. Add 10 mL antibody solution to a 10 cm petri dish and swirl to evenly coat plate.
  c. Incubate the panning plate at 4°C overnight.
2. Coat cell culture plates with PDL.
  a. For 10 cm dishes: dilute 50 µL of PDL (10 mg/mL) in 10 mL of sterile water (per dish). Add 10 mL of PDL solution to a 10 cm tissue culture plate and swirl to evenly coat plastic.
  b. For coverslips: Plate 1 coverslip in each well of a 6- or 12-well plate. Dilute 120 µL PDL solution in 12 mL of sterile water (per plate). Add 2 mL of PDL solution to each well of a 6-well plate or 1 mL to each well of a 12-well plate and swirl to evenly coat plastic.
  c. Incubate overnight at room temperature, keep sterile.
  d. Note: A higher concentration of PDL is used for glass relative to plastic as OPCs are more readily adherent to plastic cell culture dishes. It is not necessary to wash the coverslips listed above with ethanol before or after coating.
3. Prepare 0.2% BSA solution.
  a. Dilute 2 mL of 4% BSA in 38 mL of D-PBS. Store at 4 °C.
4. Prepare 0.5 mg/mL insulin solution.
5. Prepare proliferation media.
  a. Note: As the biological activity of growth factors decreases with time, and the concentration of PDGF-AA is critical for OPC cultures and amplification of cells, media should be prepared fresh when possible. Once growth factors are added, store at 4°C for up to 7 days.

Preparation the morning of the immunopanning experiment

6. Rinse and dry PDL-coated culture dishes.
  a. Rinse each plate three time with sterile water.
  b. Leave plates open in hood for a minimum of 2 hours to air dry.
  c. Note: make sure plates are dried completely as this may impact OPC attachment (Fig. 4A).
7. Coat two 10 cm petri dishes with BSL 1 solution for negative selection.

a. Dilute 20 µL BSL 1 in 20 mL D-PBS.
b. Add 10 mL BSL 1 solution to each plate and swirl to evenly coat.
c. Note: Emery & Dugas (2013) suggest two 15 cm dishes [8]. While this is more or less equivalent to four 10 cm dishes in terms of surface area, in our experience, two 10 cm dishes limits reagent use and is sufficient as microglia cell contamination has not been a problem with our protocol.

8. Coat positive selection dish with primary antibody.

a. Rinse plate coated with secondary antibody (prepared in step 1) three times with PBS. Do not let plate cell culture surface dry out after coating.
b. Prepare 40 µL rat anti-PDGFRα antibody in 10 mL of 0.2% BSA D-PBS solution.
c. Coat plate with primary antibody solution and let sit at room temperature until use, or for a minimum of two hours.

9. Prepare solutions for papain digestion, immunopanning and trypsinization.

a. For 10X ovomucoid solution, combine:
  i. 1 mL of 10X ovomucoid stock with 9 mL D-PBS.

b. For 6X ovomucoid solution, combine:
  i. 1 mL of 6X ovomucoid stock with 5 mL D-PBS.

c. For immunopanning buffer, combine:
  i. 13.5 mL D-BPS, 1.5 mL 0.2% BSA D-BPS, and 150 µL of 0.5 mg/mL insulin.

d. For 30% FBS, combine:
  i. 3 mL heat-inactivated FBS and 7 mL D-BPS.

10. Equilibrate EBSS in 37°C incubator.

a. Dilute 800 µL 10X EBSS in 7.2 mL sterile water and incubate in 10% CO_2_ incubator until use.

**Fig. 1:**
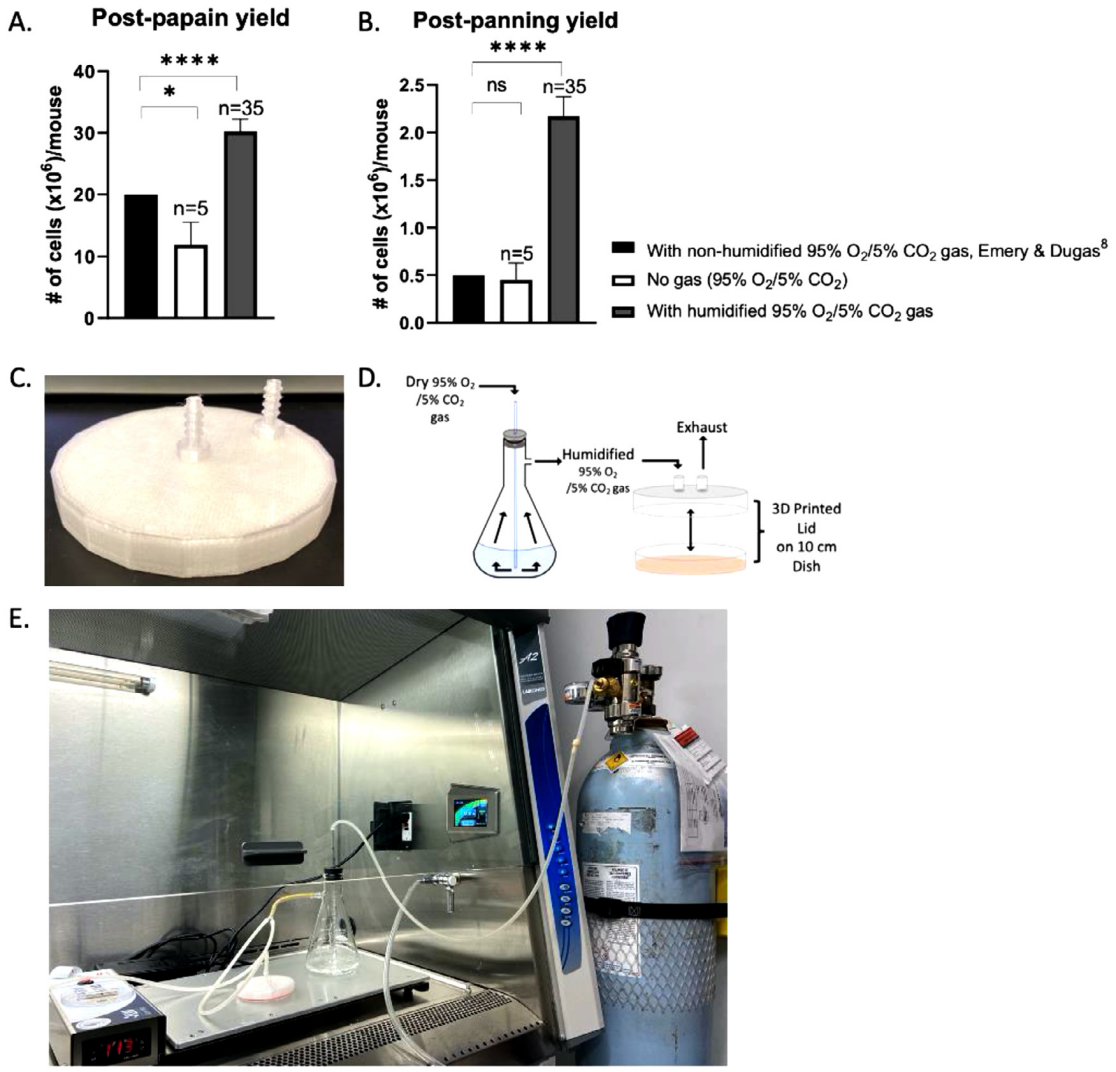
Improved cell yields and final OPC yields with optimized papain digestion**.** A. Quantification of total cell yields (expressed as x10^6^/mouse brain) following papain digestion in the Emery & Dugas (2013) protocol with non-humidified 95% O_2_/5% CO_2_ gas[8], without gas (95% O_2_/5% CO_2_) (*n* = 5) and with humidified 95% O_2_/5% CO_2_ gas (*n* = 35). B. Quantification of OPC yields (expressed as x10^6^/mouse brain) following papain digestion in the Emery & Dugas (2013) protocol with non-humidified 95% O_2_/5% CO_2_ gas[8], without gas (95% O_2_/5% CO_2_) (*n* = 5) and with humidified 95% O_2_/5% CO_2_ gas (*n* = 35). Single sample *t*-test. Data represented as mean ± SEM. C. Image of the petri lid used for perfusion. D. Schematic of the 95% O_2_/5% CO_2_ gas perfusion set-up. E. Image of the 95% O_2_/5% CO_2_ gas perfusion set-up.

11. Equilibrate papain buffer.

a. Set-up hot plate in BSC.
  i. The hot plate should be set so that papain digestion can occur at 34°C.
  ii. Note: The hot plate will likely have to be set at a higher temperature to maintain the buffer at 34°C during dissociation, for reference ours is set to 44°C. Before carrying out the protocol, test your hot plate with some papain buffer and a thermometer to determine an appropriate temperature.

b. Add 10 mL papain buffer to petri dish, place on hot plate and cover with petri lid.
c. Set-up the humidified 95% O_2_/5% CO_2_ gas perfusion system (see Fig. 1D,E).
  i. Add around 100 mL of sterile water to a 500 mL vacuum flask.
  ii. Add a stopper to the top of the flask with a 2 mL pipette through it.
  iii. Connect 95% O_2_/5% CO_2_ gas tank to 2 mL pipette with tubing.
  iv. Connect outlet of flask to petri lid with tubing.

d. Add 200 U of papain-to-papain buffer.
e. Add 200 µL of DNase I solution (2500 U) to papain buffer.
f. Add 200 µL of 10 mg/mL l-cysteine solution to papain buffer.
  v. Emery & Dugas (2013) recommend adding 2 mg of l-cysteine [8]. As weighing out 2 mg can be tedious and inaccurate, we opt to aliquot 10 mg and, when ready to use, resuspend in 1 mL of sterile water. Discard any extra.

g. Leave the papain mixture on the hot plate with humidified gas flow for 5 to 10 min to activate the papain before adding brain tissue.
  vi. Note: This optimized papain tissue digestion with humidified 95% O_2_/5% CO_2_ gas perfusion is crucial to the final OPC yield (Fig. 1B).

Dissection of the mouse brain (we typically use three P6 mice per experiment)

12. Add EBSS to a petri dish and place on ice.
13. Cryo-anesthetize and decapitate postnatal day 6 (P6) mice.
14. Starting at the vertex, cut the skin along the midline, removing it to expose the skull.
15. Starting at lambda, cut along the sagittal suture of the skull towards the nasal bone.
16. Using forceps, peel back the skull to reveal the brain.
17. Gently use forceps to cut any underlying nerves and remove the brain.
18. Dissect desired brain regions.
  a. Note: For standard cultures, yield is best when both telencephalon and mesencephalon are included in the dissociation (i.e., remove olfactory bulbs, cerebellum, pons, and any residual spinal cord)
19. Place the dissected brain in a 15 mL conical tube with EBSS while dissecting the remaining mice.
  a. Note: Work carefully but quickly during the dissection as this improves cell viability.

Papain digestion

20. Add dissected brains to papain buffer and chop, with a razor blade or scalpel, into small chunks (approximately 1 mm^3^ in size).
21. Incubate the tissue in papain solution, under humidified 95% O_2_/5% CO_2_ gas perfusion for 90 min, gently agitating the plate every 30 min.
  a. The use of humified 95% O_2_/5% CO_2_ gas perfusion at this step is critical to limit evaporation of the papain buffer.
22. Just before the 90-minute mark, add 100 µL DNase I (1250 U) to the 10X ovomucoid solution (prepared in step 9).
23. At the end of the 90 min, when the digestion is complete, transfer the papain buffer with digested brain tissue into a 15 mL conical tube and add 1 mL of 10X ovomucoid solution (with DNase I).
24. Pellet the solution for 5 min at 220 xg at room temperature.
  a. This modification adds negligible time but increases yield and minimizes the risk of aspirating tissue, compared to leaving the tissue to settle.
25. Carefully aspirate the supernatant.
26. Add 1.5 mL of 10X ovomucoid solution to digested tissue and triturate slowly (set pipette gun to low speed) with a 5 mL pipette by pipetting up and down, seven times.
27. Let tissue settle and transfer supernatant to a new 15 mL conical tube.
28. Repeat steps 26–27 once with the remaining tissue.
29. Add 1 mL of 10X ovomucoid solution to digested tissue and triturate with a 1000 µL pipette tip, by pipetting up and down, seven times.
30. Let tissue settle and transfer supernatant to a new conical tube.
31. Repeat steps 29–30 with the remaining tissue, until all 10X ovomucoid solution is used.
32. Pellet cells by centrifugation for 15 min at 220 xg (room temperature).
33. Aspirate supernatant and resuspend pellet in 6 mL of 6X ovomucoid solution (prepared in step 9) by gently pipetting up and down once.
34. Pellet cells by spinning for 15 min at 220 xg (room temperature).
35. Aspirate the supernatant and resuspend pellet in 6 mL immunopanning buffer (prepared in step 9).
36. Prewet 40 µM cell strainer with 2 mL immunopanning buffer.
37. Filter the cell solution in panning buffer and rinse filter with the remaining 7 mL of immunopanning buffer.
  a. At this point, collect 10 µL of the cell suspension for a post-papain cell count. The expected yield is 30.25 × 10^6^ cells/brain (Fig. 1A).

Panning steps

38. Immediately before use, rinse one BSL 1 coated plate three times with D-PBS.
  a. Note: Ensure panning plates are never allowed to dry.
39. Add filtered cell suspension to the first BSL 1 dish, rinsed in Step 38.
40. Incubate plate at room temperature for 15 min, gently agitating the plate every 5 min.
41. Immediately before use, rinse the second BSL 1 dish three times with D-PBS.
42. Gently shake the plate to loosen non-adherent cells and transfer non-adherent cells to the second BSL 1 plate.
43. Repeat step 40.
44. Immediately before use, rinse PDGFRα antibody-coated plate three times with D-PBS.
45. Gently shake the plate to loosen non-adherent cells and transfer non-adherent cells to a PDGFRα antibody-coated plate.
46. Incubate the plate at room temperature for 45 min, with gentle agitation every 15 min.
47. At the end of the 45 min, rinse the plate three times with D-PBS.
  a. While seemingly trivial, the modification to reduce washes from the Emery & Dugas (2013) protocol is key for the isolation of PDGFRα+ cells [8]. The interaction between OPCs and the anti-PDGFRα plate is relatively weak – if washing steps are not done gently, cells will detach and can be completely lost at this step.
  b. When washing, set pipette to gravity and pipette onto the same area of petri dish with every wash, swirl once very gently, pipette off. Do not pour D-PBS on and off the plate.
  c. It is more beneficial to minimize washes than to be too stringent about non-OPC cell contamination. The media used to culture the cells is pro-oligodendrocyte lineage and contamination of other glial cell types, such as residual astrocytes, is of minimal concern.

Trypsinization and plating

48. Add 400 µL of trypsin to the equilibrated 8 mL 1X EBSS (prepared in step 10).
49. Add the trypsin solution to cells on the PDGFRα coated plate.
50. Incubate the plate at 37°C, in a cell culture incubator, for 6 to 8 min.
51. Stop the trypsin reaction by adding 30% FBS solution.
52. Dislodge and collect the cells in a conical tube.
53. Spin for 15 min at 220 xg, room temperature.
54. Resuspend the cells in 1 mL proliferation media and count.
  a. The expected yield is 2.17 × 10^6^ cells/brain (Fig. 1B).
55. Plate 500 000 cells per 10 cm culture dish.
  a. Note: Cells will proliferate rapidly. It is ideal to start with a low cell density, so the cells do not become too confluent.
  b. Emery and Dugas (2013) suggests adding cells in a small suspension volume, spreading with a sterile glass spreader and incubating prior to adding media, in order to increase cell viability [8]. In our protocol, viability of cells has not proven to be problematic and therefore we have not used this. However, one may consider doing this if experiencing low viability.
  c. Note: we consider the day of plating as day *in vitro* 0 (DIV0).
56. Culture mouse OPCs in a 10% CO_2_ incubator.
57. At 2 DIV, supplement media with PDGF-AA, bringing the final concentration to 20 ng/mL PDGF-AA.
  a. Supplementing the cells with PDGF-AA will push for an oligodendrocyte lineage culture and induce a high rate of OPC proliferation to expand cultures (Fig. 2).

**Fig. 2:**
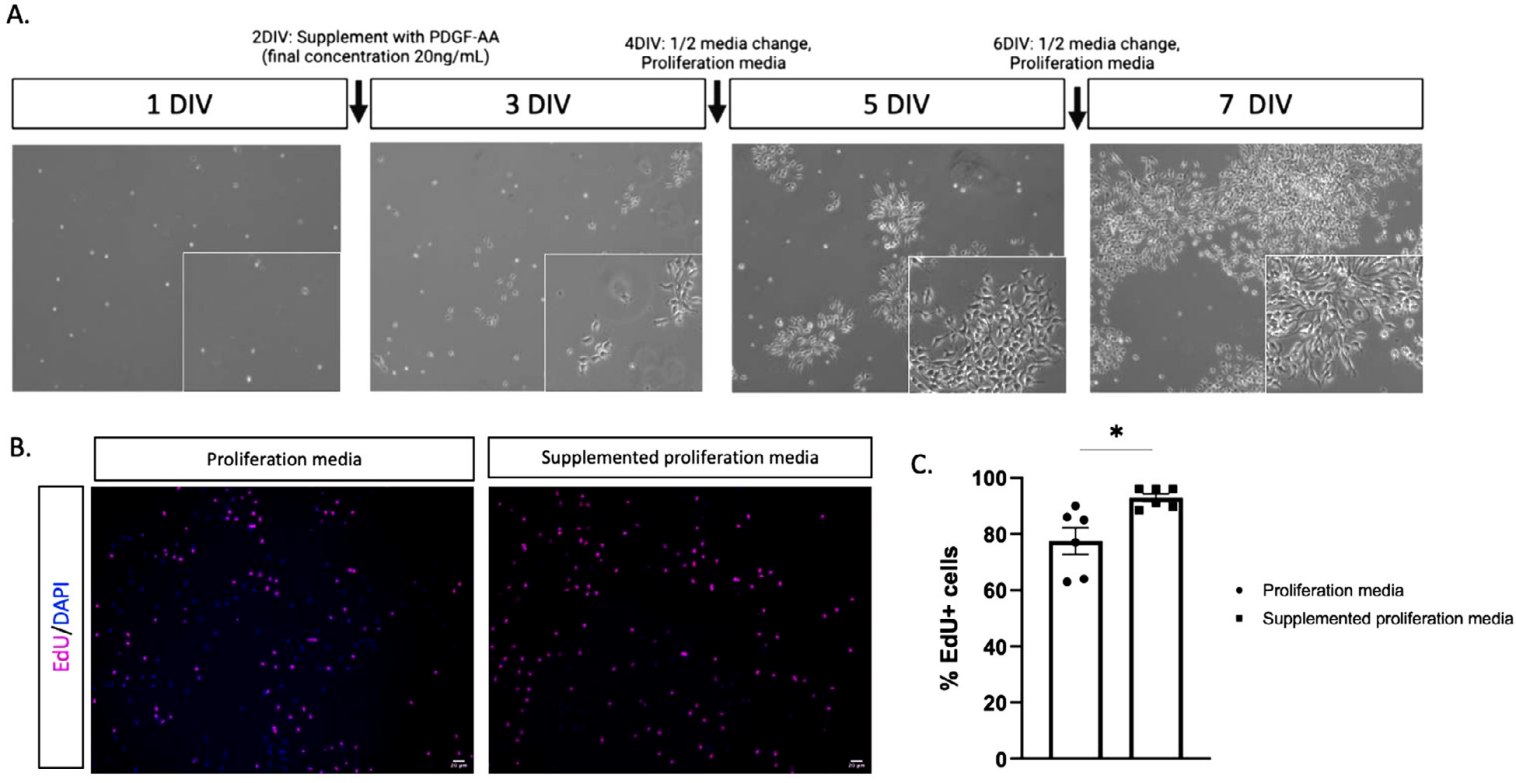
PDGFRα+ OPCs are highly proliferative**.** A. Brightfield images of OPCs in an ideal culture at 1, 3, 5 and 7 DIV and maintenance protocol (written above the images). Scale bar, 20 µm. B. Representative immunofluorescence images (from three independent experiments) of OPCs in standard proliferation media or media supplemented with PDGF-AA (final concentration: 20 ng/mL) labelled with EdU (magenta). Nuclei stained with DAPI. Images taken on an EVOS light microscope, Cy5 light cube. Scale bar, 20 µm. C. Quantification of EdU+ cells as a percentage of DAPI in standard or supplemented proliferation conditions. Data represents mean ± SEM from three independent experiments, *p < 0.05. Unpaired, two-sided Student's *t*-test.
58. At DIV4, do a half media change.
59. Continue half media changes every two days.
60. Passage cells.
  a. We typically passage at DIV 7 into cell culture dishes for experiments, as the cells at this point have proliferated but are not so dense that they start to differentiate.
  b. If the experimental objective involves differentiation, passage cells into differentiation media. Passaging will help to ensure PDGF-AA and bFGF are removed from culturing conditions and is a preferable alternative to simply replacing media with T3-containing media.

## Reagent preparation

Papain dissociation reagents:

Papain Buffer (500 mL)

**Table:** 

EBSS Stock (10X)	50 mL
MgSO_4_	60.19 mg
Glucose	2.3 g
EGTA (1 M)	1 mL
NaHCO3 (1 M)	13 mL

Bring the volume to 500 mL with ddH_2_O and autoclave to sterilize. Store at 4°C.

EBSS (250 mL,10X stock)

**Table:** 

NaCl	17 g
KCl	1 g
NaH_2_PO_4_•H_2_O	0.35 g
Glucose	2.5 g
Phenol red (0.5%)	2.5 mL

Bring the volume to 250 mL with ddH_2_O and filter to sterilize.

L-Cysteine

Prepare 10 mg aliquots of L-cysteine and store at room temperature.

Do not dissolve in water until right before use.

10X Ovomucoid papain inhibitor (33.3 mL)

**Table:** 

BSA	0.5 g
Ovomucoid	0.5 g

Combine in D-PBS. Adjust pH to 7.4 with NaOH. Bring final volume to 33.3 mL with D-PBS. Filter and sterilize with a 0.22 µm filter. Aliquot the papain inhibitor in 1 mL aliquots and store at −20°C.

6X Ovomucoid papain inhibitor (33.3 mL)

**Table:** 

BSA	1 g
Ovomucoid	1 g

Combine in D-PBS. Adjust pH to 7.4 with NaOH. Bring final volume to 33.3 mL with D-PBS. Filter and sterilize with a 0.22 µm filter. Aliquot the papain inhibitor in 1 mL aliquots and store at −20°C.

DNase I

Combine 12 500 U of DNase I per 1 mL EBSS, on ice. Filter and sterilize. Aliquot in 300 µL aliquots and store at −80°C.

Immunopanning reagents:

BSL 1

Resuspend 5 mg BSL 1 in 1 mL of 1X HEPES buffered saline. Aliquot in 20 µL aliquots and store at 4°C.

HEPES buffered saline (HBS) (100 mL, 2X stock)

**Table:** 

NaCl	0.8 g
Na_2_HPO_4_	21 mg
HEPES	0.283 g

Combine in ddH_2_O and bring pH to 8.5 with NaOH.

Then add in:

**Table:** 

CaCl_2_	1.12 mg
NaN_3_	40 mg

Bring final volume to 100 mL and filter-sterilize.

Tris–HCl (1 L, 50 mM)

Combine 7.88 g of Tris–HCl with ddH_2_O. Bring pH to 9.5 with NaOH. Autoclave to sterilize and store at 4°C.

BSA (50 mL, 4%)

Dissolve 2 g of BSA in 50 mL D-PBS. Adjust pH to 7.4 with NaOH. Filter through a 0.22 µm filter. Aliquot in 1 mL aliquots and store at −20 °C.

Trypsinization reagents:

Trypsin

Dissolve 50 000 U/mL in EBSS.

Trypsin should be aliquoted (400 µL) and stored at −80°C to prevent self-cleavage [9].

FBS

Heat shock FBS in a water bath set to 56°C for 1 hour. Aliquot and store at −20°C.

Cell culture reagents:

Poly-D-Lysine (PDL) hydrobromide (10 mg/mL)

Dissolve 100 mg of PDL in 10 mL of 1X borate buffer.

Aliquot in 250 µL aliquots and store at −20°C.

Borate buffer (400 mL)

**Table:** 

H_3_BO_3_	1.24 g
Na_2_B_4_O_7_	1.9 g

Dissolve in ddH_2_O, bring the pH to 8.5 and the volume to 400 mL with ddH_2_O.

SATO Supplement (20 mL)

**Table:** 

BSA	200 mg
Transferrin	200 mg
Putrescine	32 mg
Progesterone stock	5 µL
Sodium selenite stock	200 µL

Bring to a total volume of 20 mL with DMEM. Filter with a 0.22 µM filter to sterilize. Aliquot in 500 µL aliquots and store in −20°C.

Progesterone stock solution (make fresh when preparing SATO reagent)

Combine 5 mg of progesterone in 200 µL ethanol to make the progesterone stock solution.

Sodium selenite stock solution (make fresh when preparing SATO reagent)

Combine 4 mg of sodium selenite, 10 µL of 1 N NaOH solution and 10 mL DMEM to make the sodium selenite stock solution.

Insulin (0.5 mg/mL)

Dissolve 5 mg of insulin with 10 mL of sterile water and 50 µL of 1 N HCl. Mix and filter through a 0.22 µM filter. Store at 4°C. Remake stock every 4 weeks.

N-acetyl-L-cysteine

Dissolve 25 mg of N-acetyl-L-cysteine in 5 mL DMEM for a concentration of 5 mg/mL. Aliquot in 50 µL aliquots and store at −20°C.

Forskolin (4.2 mg/mL)

Dissolve 50 mg forskolin in 1 mL of sterile DMSO. Add another 11 mL DMSO to bring the volume to 12 mL for a final concentration of 4.2 mg/mL. Aliquot in 50 µL aliquots and store at −20°C.

Biotin (50 µg/mL)

To prepare a 10 µM stock solution, dissolve 100 mg of biotin in 40 mL of DMEM.

To prepare the final solution, dilute 20 µL of the 10 µM stock solution in 980 µL DMEM.

Aliquot in 10 µL aliquots and store at −80°C.

PDGF-AA (10 µg/mL)

Dissolve 50 µg PDGF-AA in 5 mL 0.2% BSA D-PBS. Aliquot in 50 µL aliquots and store at −80°C. bFGF (10 µg/mL)

Dissolve 50 µg bFGF in 5 mL of 0.2% BSA D-PBS. Aliquot in 50 µL aliquots and store at −80°C.

T3 (4 µg/mL)

To prepare a 20 mg/mL stock solution, dissolve 100 mg of T3 in 5 mL DMSO.

To prepare a 400 µg/mL solution, dilute 100 µL of the above 20 mg/mL solution in 4.9 mL DMEM.

To prepare the final solution, dilute 50 µL of the 400 µg/mL solution in 4.95 mL DMEM.

Aliquot in 500 µL aliquots and store at −80°C.

For 50 mL of Proliferation media, combine:

**Table:** 

Dulbecco's Modified Eagle's Medium (DMEM)	49 mL
B-27 (-vitamin A)	1 mL
Penicillin-Streptomycin	500 µL
GlutaMAX	500 µL
Sodium Pyruvate	500 µL
Insulin (0.5 mg/mL)	500 µL
SATO supplement	500 µL
Trace Elements B	50 µL
N-acetyl-l-cysteine	50 µL
Forskolin	50 µL
Biotin	10 µL
PDGF-AA	50 µL
bFGF	50 µL

Filter through a 0.22 µm filter.

Note: Emery & Dugas (2013) add CNTF and NT-3 growth factors to the media [8]. CNTF has been shown to direct OPCs toward astrocyte differentiation and we therefore omit it from the media [4]. Additionally, NT-3 has been suggested to induce differentiation of OPCs cultured on poly-lysine plates into OLs, hence we do not use NT-3 as a growth factor in our cultures [10]. Of note, while the original protocol [8] suggested the addition of B-27 in media for improved viability, it is not part of their original base growth media. In comparison, we always include B-27 in our media and specifically, use a B-27 without vitamin A as retinoic acid, a metabolite of vitamin A, has been found to promote differentiation of OPCs into oligodendrocytes [11].

For 50 mL differentiation media:

Omit PDGF-AA and bFGF and rather, include 500 µL of 4 µg/mL T3.

## Method validation

To validate our protocol, we compared the total cell yields following papain digestion and the OPC yield after immunopanning, in the absence or presence of humidified 95% O_2_/5% CO_2_ gas perfusion during the enzymatic tissue digestion, to the expected yields of the Emery & Dugas (2013) paper (Fig. 1A, B) [8]. Omitting the dry 95% O_2_/5% CO_2_ gas used in Emery and Dugas [8] resulted in a drop in yield at the post-papain count (*p = 0.0309) but to our surprise, provided comparable OPC yields (n.s., *p* = 0.0657). In comparison, our perfusion method led to a significant improvement in yield at both the post-papain step (****P<0.0001) as well as a the final OPC yield (****P<0.0001). This finding suggests an optimized papain digestion impacts the yield after the papain step and has a significant influence on the final OPC yield.

Moreover, isolation at the PDGFRα+ precursor stage means the cells are highly proliferative when maintained in culture, overcoming the often-inherent challenge of limited cell yield for primary cell culture work (Fig. 2A). In line with this, high percentages of cells were positive for EdU in an EdU uptake experiments using standard proliferation media (avg. 77.5% EdU+, following 20 hours of 10 µM EdU exposure) and this proportion was significantly enhanced when the cells were maintained using media supplemented with PDGF-AA (*p = 0.011; final PDGF-AA concentration 20 ng/mL) (Fig. 2B,C). Due to the high proliferative rate (avg. cell count 60.4 × 10^6^ cells/mouse after 7 DIV), we found that one panning dish for all immunopanning experiments, or the equivalent of up to three mouse brains, was sufficient to generate large quantities of cells, while limiting the use of reagents. The extensive proliferative capacity of early PDGFRα+ OPCs is relevant to the amplification of OPCs from transgenic mice, where the availability of mice with the desired genotype limits source tissue quantities.

**Fig. 3:**
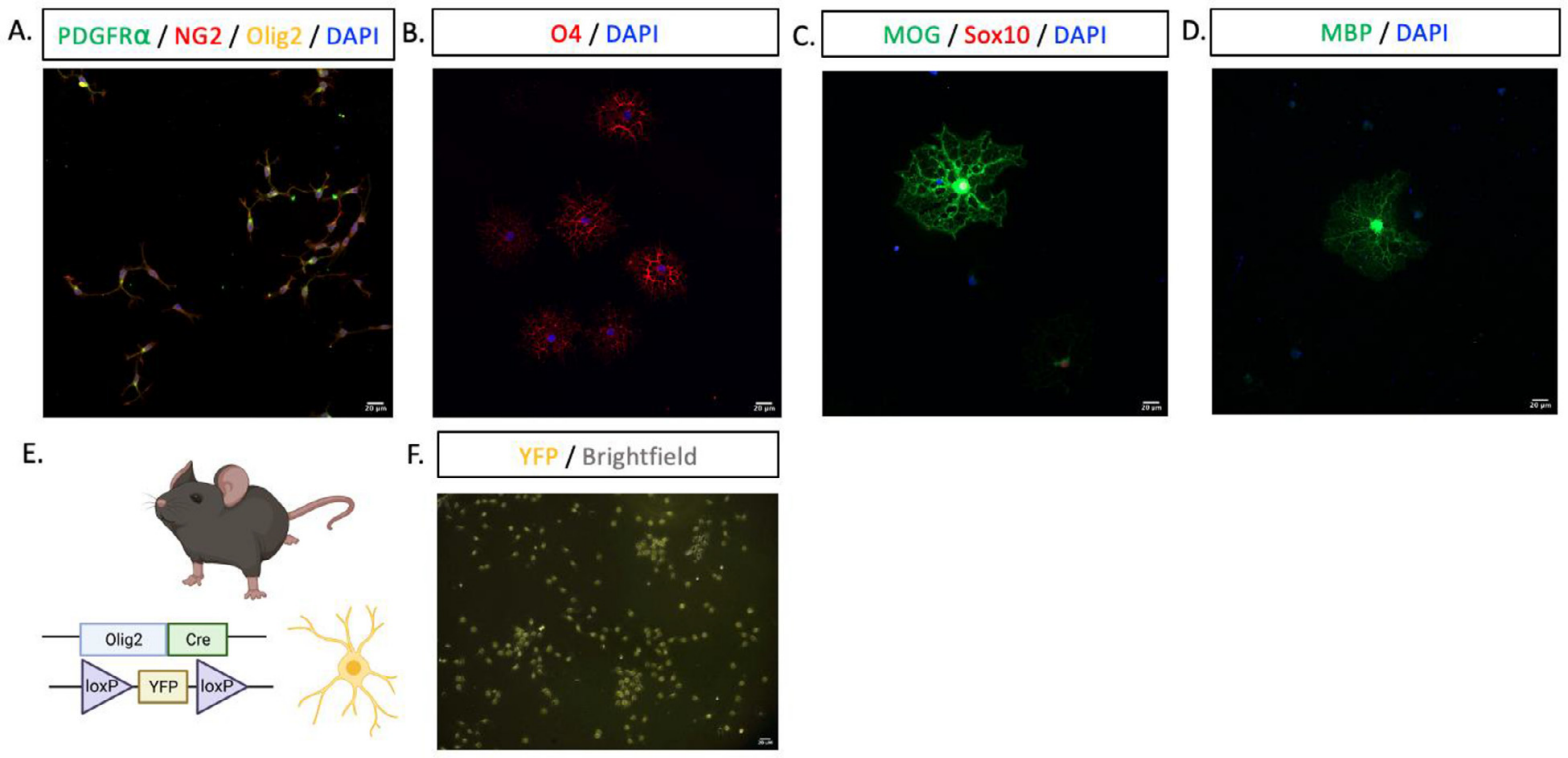
Immunocytochemistry characterization of cultures and purification from Olig2-Cre YFP+ mice. A. OPCs at 7 DIV under proliferative conditions positively labelled for PDGFRα, NG2 and Olig2, B. Immature oligodendrocytes after 2 DIV in differentiation/T3-containing media stained for O4. C. Oligodendrocytes after 3 DIV in differentiation/T3-containing media stained for MOG and Sox10 or D. MBP. E. Olig2-Cre YFP+ transgenic mouse. F. OPCs from Olig2-Cre YFP+ transgenic mice in culture (2 DIV, EVOS light microscope, YFP light cube) Antibodies: rat anti-PDGFRα (BD Pharmigen, 1:100), rabbit anti-NG2 (Milipore, 1:200), goat anti-Olig2 (R&D, 1:200), mouse anti-O4 (R&D, 1:200), goat anti-Sox10 (R&D, 1:250) rat anti-MOG (R&D, 1:300), rat anti-MBP (Novus, 1:500), AlexaFluor 488 donkey anti-rat (ThermoFisher, 1:1000), AlexaFluor 647 donkey anti-rabbit (Jackson ImmunoResearch, 1:500), AlexaFluor 594 anti-mouse (Jackson ImmunoResearch, 1:500), AlexaFluor 555 donkey anti-goat (Invitrogen, 1:500). Nuclei stained with DAPI. Labeling imaged using Zeiss LSM780 confocal microscope, YFP+ cells imaged with an EVOS light microscope. Scale bar, 20 µm.

Next, to assess that the modifications to the protocol, namely decreasing the surface area used during the BSL 1 negative selection steps and reducing the number of washes between the PDGFRα+ coated panning dish and trypsinization step, did not affect the purity of cultures, we characterized the cultures using immunocytochemistry. All cells were immunolabelled for their respective lineage markers at their corresponding stage of development, including all cultured cells under proliferative conditions labeling for the OPC markers PDGFRα+ and/or NG2+, which have different but overlapping temporal expression (Fig. 3A) [12]. Additionally, when induced to differentiate by passaging into T3-containing media, cells labelled for immature oligodendrocyte markers O4 (Fig. 3B; 2 DIV in differentiation media) or mature oligodendrocyte markers MBP/MOG (Fig. 3C,D; 3 DIV in differentiation media). Further, when isolating OPCs from constitutive Olig2-Cre YFP+ mice, all cells in the cultures were YFP+, consistent with our immunocytochemical findings indicating the purity of the oligodendrocyte lineage culture, although of note, distinct populations of Olig2+ astrocytes do exist in the mouse brain (Fig. 3E,F) [13].

**Fig. 4:**
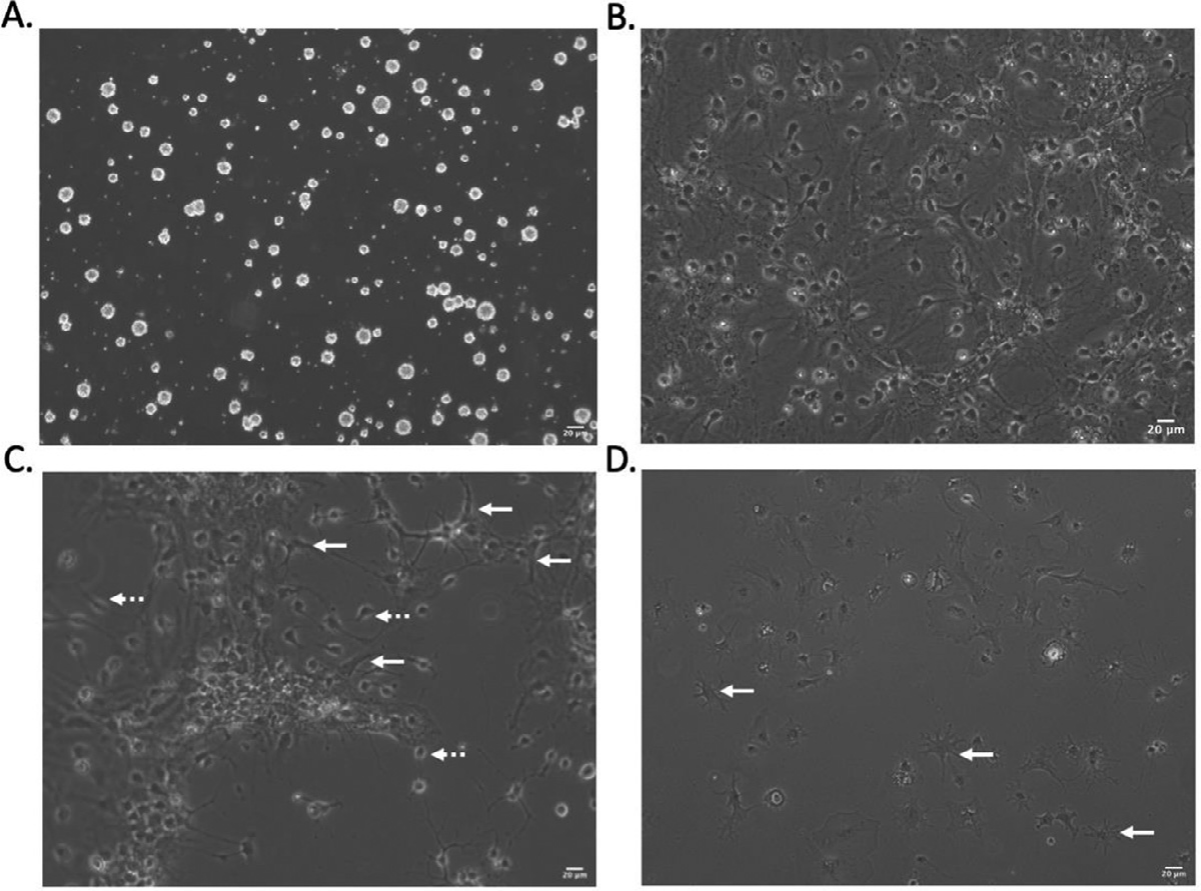
Maintenance of OPCs in culture and recognizing contaminating cell types A. PDL coating of cell culture dishes is critical for the adherence of OPCs. If the PDL coating is suboptimal, or cell culture substrates are not dried at this step, OPCs may fail to attach to the plates and subsequently form spheres, as seen here. B. In certain cases, contaminating cell types may be present in these cultures. In the case illustrated here, cells were maintained for 13 DIV under proliferative conditions but without passaging, and as a result became over-confluent, at which point cells may differentiate into astrocyte-like cells. C. Here as well, contaminating cell types (white arrows) are seen amongst the OPCs (white dashed arrows), which are themselves easily recognizable as small, rounded cells with processes. D. The media used to culture OPCs is serum-free. Inclusion of serums like Fetal Bovine Serum (FBS), such as was done in this example, directs cell fate towards the astrocyte lineage (white arrows), resulting in a mixed cell culture population. Scale bar, 20 µm.

Once isolated, OPC maintenance in culture is straightforward. Nonetheless, certain considerations should be kept in mind, for one, ensuring a proper PDL coating of the cell culture dish prior to plating. Poor PDL coating or failure to dry the plates sufficiently at this step can limit cell adherence to the cell culture substrate (Fig. 4A). Moreover, while immunopanning is advantageous compared to other isolation techniques at yielding a relatively pure OPC population, contamination by non-oligodendrocyte lineage cell types may arise in culture and are most often attributable to a high cell density, poor maintenance in pro-OPC lineage media conditions or inclusion of factors that promote the differentiation of astrocytes in the media (Fig. 4B,C,D).

**Fig. 5:**
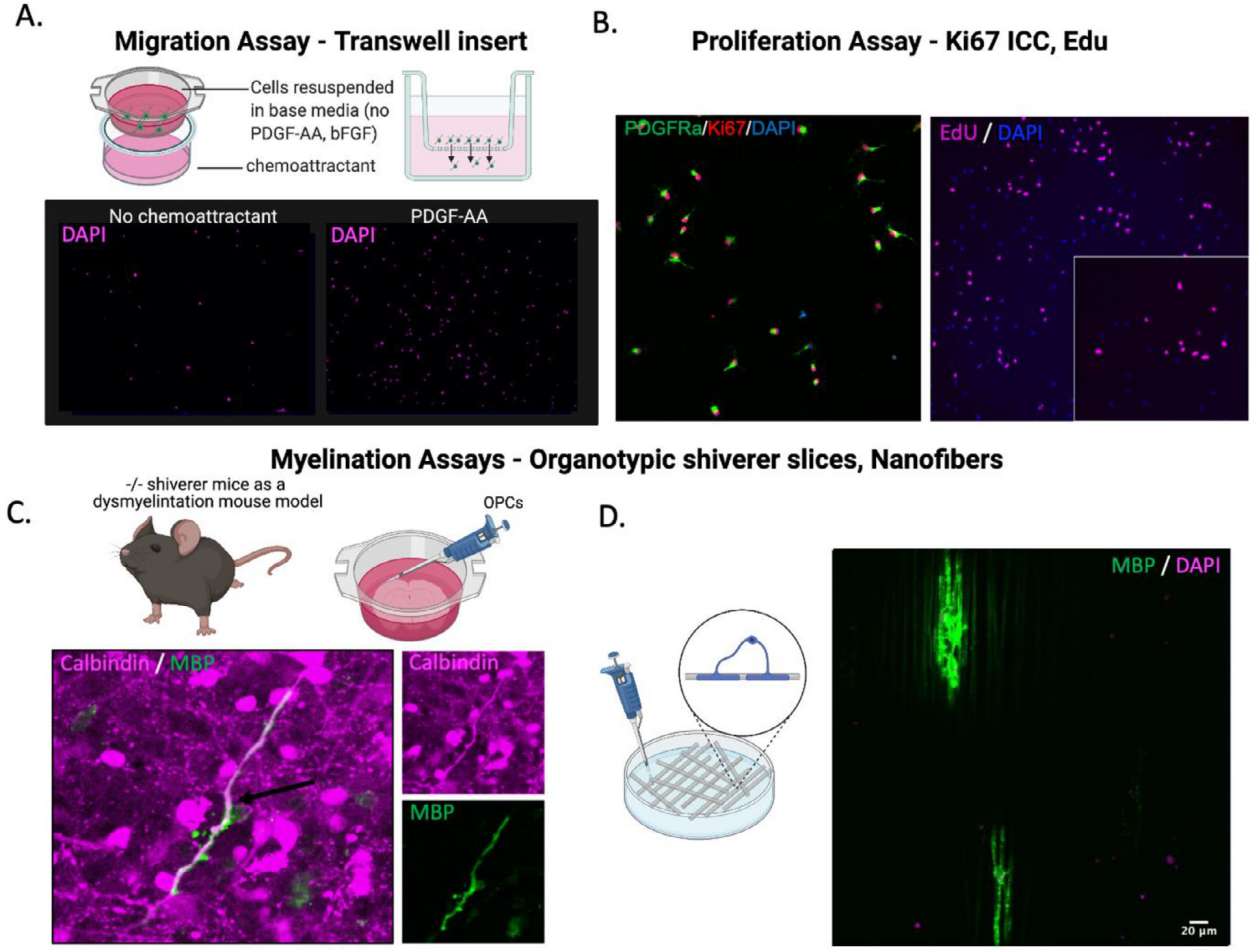
Isolation of PDGFRa+ OPCs enables the study of oligodendrocyte development. A. Transwell insert migration assay schematic and assessment of OPC migration in a control (no chemoattractant, intrinsic migration) versus chemoattractant (PDGF-AA, 20 ng/mL) driven migration. B. Proliferation assays that can be utilized to assess OPC proliferation include Ki67 staining and EdU uptake experiments. Once differentiated, oligodendrocytes *in vitro* can be used to assess myelination. C. Schematic of transplantation of OPCs into organotypic shiverer slice and immunofluorescence image demonstrating co-localization of MBP and calbindin, suggestive of axon ensheathment by transplanted OPCs, in shiverer slices collected at three weeks. D. Immunofluorescence image of positive MBP labeling in a nanofiber 3D cell-culture myelination assay, collected at two weeks. Antibodies: sheep anti-Ki67 (Novus, 1:200), rat anti- PDGFRα (BD, 1:100), rat anti-MBP (Novus, 1:500), rabbit anti-Calbindin (Swant, 1:5000), AlexaFluor 488 donkey anti-rat (ThermoFisher, 1:1000), AlexaFluor 594 donkey anti-sheep (Jackson ImmunoResearch, 1:500), AlexaFluor 647 donkey anti-rabbit (Jackson ImmunoResearch, 1:500).

Finally, as OPCs in culture can be subjected to various assays that recapitulate their development *in vivo*, an efficient method for isolating oligodendrocytes at the precursor stage provides a powerful tool for studying fundamental aspects of oligodendrocyte development. For example, OPCs in culture can be subjected to assays that assess their migration (e.g. Transwell insert migration assay; Fig. 5A) and proliferation (e.g. Ki67 staining, EdU uptake experiment; Fig. 5B). Moreover, upon differentiation, these cells provide a valuable tool to study myelination using organotypic shiverer slice culture techniques (Fig. 5C) and nanofiber cell culture systems (Fig. 5D) [14,15].

## Discussion

Here, we present an optimized immunopanning protocol for the isolation of PDGFRα+ oligodendrocyte precursor cells from mouse brain tissue. Despite the advent of numerous techniques to isolate oligodendrocyte lineage cells from rat, for various reasons, many of these techniques have proven to be inefficient for isolation from mouse brain tissue. While the immunopanning technique first described by Emery & Dugas has been of great value, the relatively weak binding of OPCs to the anti-PDGFRα plate requires special considerations when isolating PDGFRα+ mouse OPCs [8]. As the isolation of OPCs in the early, PDGFRα+ stage allows for the expansion of these cells in culture and for the study of oligodendrocyte lineage development *in vitro*, we present here an immunopanning protocol optimized specifically for PDGFRα-dependent selection.

Our data emphasizes the importance of the papain digestion step as critical to obtain increased cell yields. We found that enhancing the 95% O_2_/5% CO_2_ gas perfusion via implementation of 3D-printed lids custom-designed for perfusion via gas inlets and outlets and importantly, humidifying the system to limit evaporation of the papain buffer on the hot plate, improved the OPC yield dramatically. Indeed, in attempts of the protocol where we omitted humidification, we noticed that following the 90-minute incubation we had lost around 40% of our initial papain buffer solution, with a likely impact on reagent composition and potential deleterious drying-out effects on tissue. Moreover, the various other additions to the protocol have optimized a PDGFRα positive cell selection, without adding significant time to the protocol. Specifically, we noted that adding a short spin following the papain digestion, in the presence of a low concentration of ovomucoid solution, minimized cell loss following enzymatic digestion. Additionally, two BSL 1 coated 10 cm dishes (10 mL of solution/plate) was sufficient for the negative selection panning step and halved the amount of reagents required at this step. Moreover, limiting the number of washes of the anti-PDGFRα coated positive selection plate prior to trypsinization was crucial to enhance the yield derived from PDGFRα cell selection. In early attempts, we found that using the full six washes resulted in far fewer cells on the panning dish at the trypsinization step, while attempting a “pour-off/pour-on” wash method led all cells to detach and zero yield. Importantly, the reduced number of washes did not decrease purity as our immunocytochemistry characterization supports the purity of our cultures. Still, maintaining cell density and growth factors within desired ranges is required to avoid contamination by undesired cell types which occasionally arise through differentiation. Once in culture, OPC maintenance is quite straightforward though various considerations are merited, discussed herein to facilitate reproducibility.

In summary, our modified immunopanning protocol for the isolation and culturing of PDGFRα+ oligodendrocytes from mouse brain tissue presented here enables a high yield of a pure population of OPCs, enabling the study of various stages of oligodendrocyte development *in vitro* and with potential applications to studies of transgenic mouse models and white matter diseases.

## Statistical analysis

All data analysis was performed using GraphPad Prism 9. For the humidified 95% O_2_/5% CO_2_ gas perfusion comparison, statistical analysis was performed using a Single sample *t*-test to compare our value (minimum of five independent experiments) to the expected baseline value provided in the Emery & Dugas (2013) protocol [8]. Statistical significance was set at *p<0.05. Data shown as mean ± SEM.

## EdU uptake experiment

As a means to assess proliferation, transfected OPCs were treated with Click-IT™ EdU Cell Proliferation Kit (ThermoFisher) for imaging as per manufacturer's protocol. Briefly, OPCs were seeded onto 15 mm coverslips (EMS) in proliferation media with (20 ng/mL) or without (10 ng/mL) extra PDGF-AA supplementation. Forty-eight hours later, OPCs were treated with 10 µM EdU solution and incubated for another 20 hours. Following the EdU incubation, cells were fixed (4% PFA in PBS, 10 mins, RT), washed (3% BSA in PBS, 2 × 10 mins, RT), permeabilized (0.5% TX-100 in PBS, 20 mins, RT) and incubated with Click-IT™ reaction cocktail to detect EdU labeling (30 mins, RT). After washing, cells were stained with DAPI and mounted with Immu-Mount (ThermoFisher)**.** Each coverslip was imaged in two randomly chosen 20x objective fields using an EVOS epifluorescent microscope. EdU and DAPI signal were counted using the analyze particles function on ImageJ software and the amount of EdU+ cells (as a percentage of DAPI) were averaged for each individual coverslip. Three independent experiments were performed for all conditions.

## Animal work

C57BL/6 were obtained from Charles River Laboratory Canada. *Olig2-cre* mice were obtained from Jackson laboratories (#025567) and backcrossed onto a C57BL/6 background. R26-stop-YFP mice were a generous gift from Dr. William Richardson (University College London) [16].

## Ethics statements

All procedures with animals were approved by the Animal Resource Division of the RI-MUHC (protocol # 2018–8055) and performed in accordance with the Canadian Council for Animal Care Guidelines for animal use in research.

## CRediT authorship contribution statement

**Julia Macintosh:** Conceptualization, Methodology, Investigation, Writing – original draft, Writing – review & editing. **Mackenzie A. Michell-Robinson:** Conceptualization, Methodology, Writing – review & editing. **Xiaoru Chen:** Methodology, Writing – review & editing. **Daryan Chitsaz:** Methodology, Writing – review & editing. **Timothy E. Kennedy:** Methodology, Writing – review & editing. **Geneviève Bernard:** Conceptualization, Methodology, Writing – original draft, Writing – review & editing, Supervision.

## Declaration of Competing Interests

The authors declare that they have no known competing financial interests or personal relationships that could have appeared to influence the work reported in this paper.

## Data Availability

Data will be made available on request.
